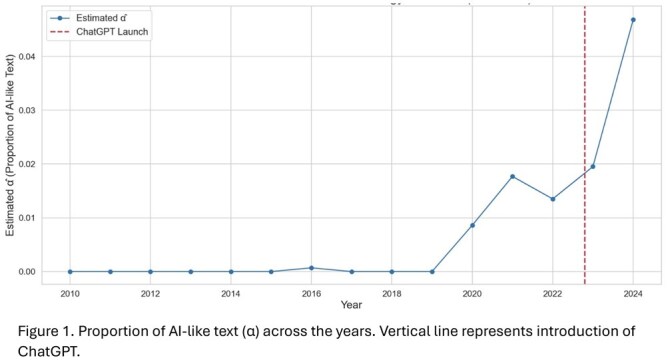# Poster Session I - A52 THE USE OF LARGE LANGUAGE MODELS IN GASTROENTEROLOGY LITERATURE: A GROWING ARTIFICIAL INTELLIGENCE FOOTPRINT

**DOI:** 10.1093/jcag/gwaf042.052

**Published:** 2026-02-13

**Authors:** A Zoughlami, A Arezki, E Medawar, S Arezki, T Bessissow

**Affiliations:** Division of Gastroenterology, McGill University Health Centre, Montreal, QC, Canada; Division of Urology, McGill University Health Centre, Montreal, QC, Canada; Division of Gastroenterology, University of Ottawa, Ottawa, Canada, Ottawa, ON, Canada; McGill University School of Computer Science, Montreal, QC, Canada; Division of Gastroenterology, McGill University Health Centre, Montreal, QC, Canada

## Abstract

**Background:**

The integration of Large Language Models (LLMs) into academic writing has transformed scientific literature, but the adoption within the gastrointestinal (GI) literature remains to be quantified.

**Aims:**

In this study, we aim to estimate the proportion of classical LLM-related language in GI abstracts from 2010 to 2024, and to characterize its variation across impact factor (IF) quartiles, and impact on lexical patterns.

**Methods:**

We conducted a retrospective, bibliometric analysis of 158,473 PubMed-indexed GI abstracts, sourced from all GI-related journals with 2024 IF ≥ 2 as found on Clarivate. A synthetic corpus of 10,000 GPT-3.5 generated abstracts was used to model AI-like linguistic distributions. The annual proportion of AI-like text (α) was estimated using a maximum-likelihood mixture model with Laplace smoothing. Journals were stratified into quartiles by their 2024 IF for sub-analysis. Lexical diversity was quantified on a yearly basis, using the type-token ratio (TTR).

**Results:**

Between 2010-2019, α was negligible (<0.001%), with only a discrete inflection beginning in 2016 (α = 0.07%). A sharp rise is noted after the introduction of ChatGPT, reaching successively 0.86% (2020), 1.77% (2021), 1.35% (2022), 1.95% (2023), and finally up to 4.68% (2024). By IF quartile, α demonstrated a U-shaped curve, lowest in Q2/Q3 (4.2% and 2.97% respectively), and highest in Q1/Q4 (5.4% and 5.7%), suggesting disproportionate adoption in both high and lower-impact journals. Lexical diversity remained stable throughout a measured period of 15 years (TTR range 0.0067 to 0.0089), demonstrating that the increased AI-related language was not associated to measurable shifts in vocabulary.

**Conclusions:**

The prevalence of LLM-related language in GI abstracts has increased sharply since the mass-introduction of LLMs, with a five-fold surge subsequent to the release of ChatGPT. These findings suggest a growing integration of LLMs in the GI body of knowledge, suggesting a need for clear editorial policies and standards of transparency regarding AI-assisted writing.

**Funding Agencies:**

None